# Role of oral gonadotropin-releasing hormone antagonists in the management of uterine adenomyosis: a narrative review

**DOI:** 10.1530/RAF-26-0075

**Published:** 2026-09-02

**Authors:** Jacques Donnez, Olivier Donnez, Hugh S Taylor, Felice Petraglia, Andrew W Horne, Christian M Becker, Stefan P Renner, Marionna Rius, Elke Bestel, Christina Anna Stratopoulou, Marie-Madeleine Dolmans

**Affiliations:** ^1^ Department of Gynecology, Université Catholique de Louvain, Brussels, Belgium; ^2^ Department of Gynecology, Société de Recherche pour l’Infertilité (SRI), Brussels, Belgium; ^3^ Centre de l’Endométriose Complexe, Chirurgie Endoscopique Pelvienne, Polyclinique Urbain V (ELSAN Group), Avignon, France; ^4^ Department of Obstetrics, Gynecology and Reproductive Sciences, Yale School of Medicine, New Haven, Connecticut, USA; ^5^ Obstetrics and Gynecology Unit, Department of Clinical Experimental and Biomedical Sciences, University of Florence, Florence, Italy; ^6^ EXPPECT Edinburgh Centre for Reproductive Health, University of Edinburgh, Edinburgh, UK; ^7^ Endometriosis CaRe Centre, Nuffield Department of Obstetrics and Gynaecology, University of Oxford, Oxford, UK; ^8^ Department of Gynecology and Obstetrics, Hospital Böblingen, Sindelfingen, Germany; ^9^ Endometriosis Unit, Hospital Clinic of Barcelona, University of Barcelona, Barcelona, Spain; ^10^ Department of Medical Affairs, Theramex, London, UK; ^11^ Gynecology Research Laboratory, Institut de Recherche Expérimentale et Clinique, Department of Gynecology, Université Catholique de Louvain, Brussels, Belgium; ^12^ Gynecology Department, Cliniques Universitaires St-Luc, Brussels, Belgium

**Keywords:** adenomyosis, pain, heavy menstrual bleeding, quality of life, linzagolix, elagolix, relugolix, GnRH antagonist

## Abstract

**Abstract:**

In women with uterine adenomyosis, bleeding and pain can have a major impact on quality of life. There is clearly a widespread demand for effective long-term medical therapies. The objective of this narrative review was to investigate the efficacy of oral gonadotropin-releasing hormone (GnRH) antagonists in the management of uterine adenomyosis by analyzing data from two *post hoc* analyses of two prospective randomized trials (elagolix and relugolix), one retrospective study (relugolix) and one prospective study (linzagolix). All three GnRH oral antagonists were able to control the disease and uterine fibroid-related heavy menstrual bleeding, enhancing quality of life. When administered without add-back therapy, high doses of GnRH antagonists were found to significantly and rapidly reduce uterine volume and the severity of adenomyotic lesions. Nevertheless, some limitations should be addressed. The *post hoc* analyses were a retrospective look at two prospective randomized clinical trials. In the elagolix study, most women underwent both transvaginal ultrasound and magnetic resonance imaging (MRI). Some who opted out of MRI may have had concurrent adenomyosis that was undetected by ultrasound, so they were not included in the adenomyosis subset. In the relugolix study, baseline ultrasounds were not specifically performed to spot adenomyosis that may have been overlooked in case of large fibroids, as shadowing can partially or completely obscure the disease. While the linzagolix study is prospective and evaluates data from women with proven adenomyosis documented by MRI, the number of cases is limited and randomized controlled trials are needed to confirm and validate this concept.

**Lay summary:**

Uterine adenomyosis is characterized by the presence of endometrial glands inside the myometrium, the muscular layer of the uterus. Transvaginal ultrasound and magnetic resonance imaging now facilitate its diagnosis. The estimated prevalence of adenomyosis among patients of reproductive age is 20–30%. Rising rates in younger patients combined with a growing trend to postpone first pregnancy underscore the importance of understanding the mechanisms behind its development. We need novel strategies for the treatment of typical but distressing symptoms of adenomyosis, which include pelvic pain, abnormal uterine bleeding, menstrual cramps, painful sex and infertility. There is a lack of approved pharmacological agents specifically indicated for the treatment of adenomyosis, presenting a clear unmet need in this population. Oral gonadotropin-releasing hormone (GnRH) antagonists have recently emerged as a promising new therapeutic option. Their advantages over GnRH agonist depot formulations include an absence of the flare-up effect, hence avoiding initially worsening symptoms and rapid reversibility.

## Introduction

Adenomyosis is a condition where the lining of the womb starts growing into the muscle of its outer wall. In the past, diagnosing the disease was largely based on data from histological specimens, typically after hysterectomy. It is being characterized by endometrial tissue inside the myometrium to a depth of at least 2.5 mm, often surrounded by hyperplastic and hypertrophic smooth muscle (Donnez *et al.* 2018, García-Solares *et al.* 2018). Today, improvements in imaging techniques, such as transvaginal ultrasound (TVUS) and magnetic resonance imaging (MRI), facilitate its diagnosis, yielding major advances in the field (Van den Bosch *et al.* 2015, Bazot & Daraï 2018, Chapron *et al.* 2020, Guo 2020, Habiba *et al*. 2020, Donnez *et al.* 2021*a*, Stratopoulou *et al.* 2021*a*,*b*, 2023). The emphasis is increasingly shifting to the endomyometrial zone (Stratopoulou *et al.* 2021*b*, Gordts *et al.* 2023), where mast cells and M2 macrophages have been identified and are thought to play a key role in the invasion of the myometrium by endometrial cells (Binda *et al.* 2017, Stratopoulou *et al.* 2021*a*, 2023, Wacheul *et al.* 2025).

The estimated prevalence of adenomyosis among patients of reproductive age is 20–30% (Vercellini *et al.* 2006, Parazzini *et al.* 2009, Naftalin *et al.* 2012), and its association with deep endometriosis is not uncommon (Donnez *et al.* 2019, Chapron *et al.* 2020). Rising rates in younger patients (Brosens *et al.* 2015, Vannuccini *et al.* 2024), combined with a growing trend to postpone first pregnancy, underscore the importance of understanding the mechanisms behind its development. Despite its prevalence and severity of symptoms, the pathogenesis of adenomyosis has not yet been elucidated. The first and foremost hypothesis posits invagination of the endometrial basalis, hyperperistalsis and activation of the tissue injury and repair (TIAR) mechanism (Leyendecker *et al.* 2009, García-Solares *et al.* 2018, Stratopoulou *et al.* 2021*c*). There is also strong evidence that estrogens play a key role (Vannuccini & Petraglia 2019, Zhai *et al.* 2020, Donnez *et al.* 2021*a*,*b*). Typical symptoms of adenomyosis include pelvic pain, abnormal uterine bleeding, dysmenorrhea, dyspareunia and infertility, but some patients can be asymptomatic (Chapron *et al.* 2020, Donnez *et al.* 2021*a,b*, 2024*a*, Vannuccini & Petraglia 2019, Vannuccini *et al.* 2025).

This narrative review focuses specifically on gonadotropin-releasing hormone (GnRH) antagonists, looking to evaluate the role of this new class of oral medication in symptomatic adenomyosis. Based on a targeted PubMed/MEDLINE literature search and review of major documents published up to May 2026, this narrative review summarizes the clinical evidence regarding GnRH antagonists (elagolix, relugolix and linzagolix) in adenomyosis. We selected original articles and *post hoc* analyses from the existing literature reporting data specifically on adenomyosis patients given GnRH antagonist therapy. Evidence from randomized controlled extension studies and emerging literature was critically reviewed, focusing on control of heavy menstrual bleeding, pain reduction, uterine volume, quality of life and the role of add-back therapy. The number of papers included was limited to *post hoc* analyses of two prospective randomized trials (elagolix and relugolix), one retrospective study (relugolix) and one prospective study (linzagolix).

This review does not include nonhormonal therapeutic alternatives. It is aimed at patients seeking conception in the future or those who have completed their family, but do not wish to undergo hysterectomy or other minimally invasive procedures, such as radiofrequencies, magnetic resonance-guided focused ultrasound or uterine artery embolization.

## Are first-line medical treatments for adenomyosis really effective?

There is a lack of approved pharmacological agents specifically indicated for the treatment of adenomyosis, presenting a clear unmet need in this population (Table 1). Certain hormones can be used off-label to alleviate symptoms, such as the levonorgestrel intrauterine system (LNG-IUS), the most widely researched for reduction in heavy menstrual bleeding (HMB) and pain. Mifepristone and dienogest have also shown some degree of efficacy (Vannuccini *et al.* 2025). New medications, such as selective progesterone receptor modulators (SPRMs), have proved unsuitable, however, as they induce endometrial changes in intramyometrial endometrium (Donnez & Dolmans 2016, Conway *et al.* 2019).

**Table 1:** Common medical treatments for adenomyosis and their advantages and drawbacks.

Medication	Advantages	Drawbacks
NSAIDs	Reduce dysmenorrhea	Less efficient than other drugs
	Nonhormonal composition, so safe for women wishing to conceive	Prolonged use linked to side effects
	May alleviate uterine hypercontractility	Inability to tackle underlying pathogenic mechanisms
		Not effective against HMB
COCs	Relatively effective at relieving pain	Doubtful effectiveness in terms of reducing HMB and uterine volume
	Better tolerability and fewer side effects than other hormonal drugs	Limited clinical data to support their use over progestin-only pills
	Lower cost	Risk of thromboembolic events
	Efficacy possibly comparable to the LNG-IUS	
Dienogest	Superior to other hormonal drugs at decreasing pain and uterine volume	High dropout rates due to adverse effects
	Reduces bleeding duration and volume	Incidents of anemia because of metrorrhagia
	Milder hypoestrogenic effects than with GnRH agonists, allowing prolonged use	Approximately a third of patients fail to respond due to progesterone resistance
LNG-IUS	Diminishes pain and HMB	Positive outcomes tend to decrease over time
	More tolerable than dienogest	Questionable effectiveness in terms of reducing uterine volume
	Can be used long term	
Etonogestrel	Adequately reduces pain and HMB	May not work against increased uterine volume
	Suitable for long-term use	Often causes spotting and weight gain
		Less efficient than other progestins (LNG-IUS and dienogest)
SPRMs	Diminish serum E2 levels	Numerous reports of exacerbation of symptoms and imaging features
	Reduce HMB	Limited to specific indications by the European Medicines Agency
GnRH agonists	Alleviate pain	Flare-up effect
	Induce amenorrhea	Severe hypoestrogenic side effects, especially loss of BMD
	Efficient at reducing uterine volume and junctional zone thickness	Long-term administration not recommended without ABT
		Random reversibility
GnRH antagonists	Rapid action and symptom relief without initial flare-up	Loss of BMD and other hypoestrogenic side effects at high doses
	Effectively reduce HMB, uterine volume and pain symptoms	Less efficient at volume reduction when combined with ABT
	Dose flexibility allows moderate E2 suppression with less severe side effects	Contraception may or may not be achieved depending on dose
	Easily reversible, suitable as pretreatment for subfertile patients	

### Nonsteroidal anti-inflammatory drugs (NSAIDs)

NSAIDs alleviate pain and inflammation, so are frequently used as the first line of treatment for dysmenorrhea, but their effect on potential underlying pathogenic mechanisms is doubtful. According to a meta-analysis of 80 trials, between 45 and 53% of patients taking NSAIDs experienced at least moderate pain relief, more than double those in the placebo group (Marjoribanks *et al.* 2015). NSAIDs may therefore be safe to use as first-line therapy for pain, but their overall efficacy in terms of managing adenomyosis-related symptoms is limited and prolonged use should be avoided because of side effects.

### Combined oral contraceptives (COCs)

For decades, COCs have been the drug of choice for management of pelvic pain associated with endometriosis and adenomyosis. According to a recent meta-analysis comparing different hormonal therapies for adenomyosis-related pelvic pain, COCs were generally found to perform similarly to the LNG-IUS after 6 months of treatment (Etrusco *et al.* 2025).

### Dienogest

There is an abundance of evidence supporting use of dienogest for symptomatic adenomyosis. According to recent meta-analyses, this medication can significantly ease dysmenorrhea, chronic pelvic pain and HMB (Ali *et al.* 2024, Lin *et al.* 2025). Comparatively speaking, dienogest appears to be better at relieving dysmenorrhea and uterine enlargement than both COCs and the LNG-IUS (Etrusco *et al.* 2025, Su *et al.* 2026). However, patients treated with dienogest may be more prone to experiencing adverse events, such as spotting or abnormal bleeding, hot flashes and breast tenderness (Etrusco *et al.* 2025, Su *et al.* 2026). Indeed, despite favorable outcomes, around 50% of patients may need to switch treatment due to persistent side effects (Chen *et al.* 2025, Vannuccini *et al.* 2025). Metrorrhagia appears to be the most common side effect of the medication, occurring in almost all patients to varying degrees of severity, and can lead to anemia in certain cases (Osuga *et al.* 2017*a*,*b*). Another disadvantage of dienogest and progestogens in general is that approximately one-third of patients fail to respond to this type of drug, likely due to progesterone resistance, thereby requiring alternative options (Vercellini *et al.* 2016, Donnez *et al.* 2021*a*, Donnez & Dolmans 2021).

### LNG-IUS

Existing data collectively endorse use of the LNG-IUS containing 52 mg levonorgestrel for relief of dysmenorrhea, HMB and enlarged uterine volume in symptomatic adenomyosis patients (Li *et al.* 2019). Compared with dienogest, the LNG-IUS may be more appropriate in the long term, as it was shown to perform equally well at managing pain and HMB. Side effect-related dropout rates were indeed much lower in this treatment group after one year (15%) vs 49% with dienogest (Vannuccini *et al.* 2025). Uterine volume is typically less affected by the LNG-IUS, which seems to be a determinant of treatment success (Lee *et al.* 2016). It actually appears that dienogest is for the most part superior for long-term management of both pain and HMB (Li *et al.* 2019, Ota *et al.* 2021).

### Subcutaneous etonogestrel implant

There are little clinical data on etonogestrel implant medication for adenomyosis management, but the results look encouraging so far. Studies suggest that etonogestrel has a positive impact on HMB and dysmenorrhea symptoms, despite uterine volume remaining unchanged (Nie *et al.* 2021, Niu *et al.* 2021). Altered bleeding patterns and weight gain were often observed among patients in all studies, but they were generally tolerable. In one study comparing etonogestrel with the LNG-IUS, the latter was deemed superior at alleviating major disease symptoms, namely dysmenorrhea, blood loss and uterine volume (Wei *et al.* 2024). All in all, etonogestrel constitutes an acceptable option for patients who prefer to refrain from oral or intrauterine progestogens, but its efficacy remains lower than other drugs in this category.

### SPRMs

Some studies have recorded exacerbation of adenomyosis after treatment with ulipristal acetate (UPA) (Conway *et al.* 2019, Donnez & Donnez 2020*a*, Calderon *et al.* 2021), including progression of imaging features of the disease and notable worsening of clinical symptoms such as pelvic pain and HMB. Moreover, the European Medicines Agency has restricted the use of UPA (Esmya) due to incidents of severe liver injury.

## Are second-line medical treatments more effective?

### GnRH agonists

GnRH agonist use in the context of adenomyosis relies on the antiproliferative effects these drugs exert within the myometrium, following the extreme drop in serum estradiol (E2) levels and subsequent amenorrhea. It has also been demonstrated that GnRH agonist therapy can normalize junctional zone thickness, one of the most typical MRI features associated with adenomyosis (Imaoka *et al.* 2002). In fact, both goserelin acetate and triptorelin acetate could prove more effective than dienogest at treating menorrhagia and uterine enlargement (Fawzy & Mezbah 2015, Ji *et al.* 2022).

The main disadvantage of GnRH agonists is the rapid and drastic loss of bone mineral density (BMD), ranging from 2 to 6% from baseline after only six months, when add-back therapy (ABT) is not administered (Sauerbrun-Cutler & Alvero 2019). Other common hypoestrogenic effects include vasomotor syndrome, mood swings and genital atrophy to varying degrees. Even though these symptoms can be partially managed by concurrent use of ABT, prolonged administration of GnRH agonists would be advised only when other medications fail and surgery is not a safe option (Vannuccini *et al.* 2018). The use of GnRH agonists should be considered as a second-line therapy and only for short-term administration because of its severe hypoestrogenic side effects (BMD loss and menopausal symptoms), initial flare-up responsible for worsening symptoms and slow reversibility. This flare-up effect and random reversibility may be regarded as disadvantages compared with GnRH antagonists (Vannuccini *et al.* 2018, Cope *et al.* 2020, Donnez *et al.* 2021*a*,*b*, Che *et al.* 2023, Ishizawa *et al.* 2023, Catherino *et al.* 2024).

### Is there a place for new options such as oral GnRH antagonists?

As adenomyosis is essentially estrogen-dependent (Donnez *et al*. 2021*a*, Stratopoulou *et al.* 2021*a*,*b*), hormone therapies reducing circulating estrogens may prevent cyclic intramyometrial growth of endometrial glands and mitigate symptoms by decreasing hyperinflammation. Oral GnRH antagonists may therefore constitute a promising new therapeutic option, allowing dose-dependent control of E2 levels (Donnez *et al.* 2017, Donnez & Donnez 2020*a*).

The aim of this narrative review was to investigate the efficacy of oral GnRH antagonists in the management of uterine adenomyosis. The number of papers included was limited to *post hoc* analyses of two prospective randomized trials (elagolix and relugolix), one retrospective study (relugolix) and one prospective study (linzagolix).

#### Elagolix

Elagolix is an oral, nonpeptide GnRH antagonist that achieves rapid, reversible and dose-dependent suppression of gonadotropins and ovarian sex steroids (Taylor *et al.* 2017, Surrey *et al.* 2018). In two identical phase 3 trials (ELARIS UF-1 and UF-2), 300 mg elagolix twice daily (BID) with hormonal ABT (1 mg E2/0.5 mg norethindrone acetate once daily (QD)) reduced HMB in women with uterine fibroids. Of 786 patients treated across the two trials, 16% (126 women) were diagnosed with uterine fibroids and coexisting adenomyosis by ultrasound (TVUS) and/or MRI (Muneyyirci-Delale *et al.* 2021).

##### HMB

In ELARIS UF-1 and UF-2 trials, women were randomized (1:1:2) at the start of the 6-month treatment period to either a placebo, 300 mg elagolix BID alone or 300 mg elagolix BID with hormonal ABT (1 mg E2/0.5 mg norethindrone acetate QD). Findings from the pooled analysis of these two identical trials demonstrated that in women with uterine fibroids and concurrent adenomyosis, elagolix with hormonal ABT was more effective at reducing HMB than the placebo, as determined by multiple endpoints. Indeed, in this subset, a significantly greater proportion of women given elagolix with ABT (77.1%) met both primary endpoint criteria (proportion of women showing less than 80 mL of menstrual blood loss (MBL) during the final month and a reduction of around 50% in MBL from baseline to the final month) than did women given the placebo (12.2%) (Muneyyirci-Delale *et al.* 2021). While not statistically compared, rates of decreased HMB in women with uterine fibroids and concomitant adenomyosis were numerically similar to those without an adenomyosis diagnosis at baseline, as well as the overall pooled study population.

##### Uterine volume

In women with adenomyosis, the mean change in uterine volume evaluated by TVUS from baseline to 6 months with elagolix plus ABT was significantly greater (−48.9 cm^3^) than with the placebo (+65.7 cm^3^). The mean change was not significant in women without coexisting adenomyosis at baseline (Muneyyirci-Delale *et al.* 2021). Interestingly, the mean change in uterine volume was more pronounced (−109.4 cm^3^) in women with concurrent adenomyosis treated with 300 mg elagolix without ABT.

##### Pain and quality of life

In the elagolix studies, subset analyses in women with fibroids and concomitant adenomyosis revealed that elagolix with ABT resulted in better quality of life than the placebo in terms of symptom severity and health-related total quality of life scores. Indeed, scores in the adenomyosis subset after elagolix with ABT were consistent with UFS-QOL (Uterine Fibroid Symptom and Quality of Life) scores validated in healthy women without fibroids (Muneyyirci-Delale *et al.* 2021).

### Conclusion

In their publication, Muneyyirci-Delale *et al.* (2021) concluded that elagolix with ABT is effective at reducing HMB in women with uterine fibroids, despite the presence of coexisting adenomyosis.

### Limitations

Most women in the trials underwent both TVUS and MRI (87%), but some who opted out of MRI may have had concomitant adenomyosis that went undetected by ultrasound. These patients were not included in the adenomyosis subset. While elagolix alone (300 mg BID) was effective at stemming HMB associated with uterine fibroids, with or without adenomyosis (Muneyyirci-Delale *et al.* 2021), it was not discussed in the manuscript. This is unfortunate, as readers were unable to gauge the efficacy of the drug without ABT. The same thing could be said of uterine volume, as GnRH antagonist therapy at high doses without ABT significantly reduces adenomyotic uterine volume, which is not the case when low doses (150 mg QD) are administered (Barseghyan *et al.* 2023).

#### Relugolix

Phase 3 LIBERTY clinical trials were designed to investigate the efficacy and safety of relugolix combination therapy in women with HMB-associated uterine fibroids. When these trials were designed, there was little recognition of adenomyosis as a separate disease, and its presence was not an exclusion criterion for participation (Al-Hendy *et al.* 2021). With growing interest in adenomyosis, *post hoc* analysis was undertaken to evaluate the prevalence of concomitant adenomyosis upon study entry. Baseline ultrasound images from the pooled LIBERTY studies were formally assessed for adenomyosis *post hoc* by two expert radiologists according to a prespecified classification derived from consensus-based MUSA criteria for the classification of adenomyosis available at that time (Catherino *et al.* 2025).

In these phase 3 LIBERTY trials, participants were randomized (1:1:2) at the start of the 6-month treatment period to either a placebo, 40 mg relugolix alone for 12 weeks followed by 40 mg with hormonal ABT (1 mg E2/0.5 mg norethindrone acetate), termed the delayed therapy group, or 40 mg relugolix with hormonal ABT (1 mg E2/0.5 mg norethindrone acetate), termed the combined therapy group. Of the 610 women who completed the study, 111 (18.2%) had a baseline diagnosis of adenomyosis, indicating that the prevalence was identical to that observed in the ELARIS UF-1/UF-2 population.

##### HMB

This *post hoc* analysis investigated the efficacy of 40 mg relugolix with ABT at reducing HMB for up to 24 weeks and concluded that decreased HMB and MBL observed in the overall LIBERTY 1/2 population were also similar in the subgroup of women with adenomyosis at baseline. In the relugolix with ABT group, the responder rate in terms of relief from HMB for women with adenomyosis and uterine fibroids (83.8%) was numerically higher than in the overall LIBERTY 1/2 population (LIBERTY 1: 73%; LIBERTY 2: 71%). The percentage change in MBL volume by week 24 was comparable between groups (85.3% in adenomyosis patients and 84.3% in the LIBERTY 1 and LIBERTY 2 populations) (Catherino *et al.* 2025).

#### Uterine volume

While no statistical comparisons were made between treatment arms or patients with adenomyosis and the overall study population, a number of trends were observed. Surprisingly, at baseline, mean indices of fibroid volume and uterine volume were lower in the subgroup of women with adenomyosis (41.41 and 303.86 cm^3^, respectively) than in the relugolix combination therapy group in the overall LIBERTY 1/2 population (LIBERTY 1: 71.9 and 379.1 cm^3^; LIBERTY 2: 73.3 and 387.7 cm^3^) (Catherino *et al.* 2025). By week 24, the least square mean uterine volume in women in the relugolix combination therapy group, delayed therapy group and placebo group had decreased by 22.2, 24.4 and 5.8%, respectively. Notably, there was no difference between the delayed therapy group and combined therapy group at week 12, although a significant difference could be expected in women given relugolix alone.

##### Pain and quality of life

Similar improvements in health-related quality of life were found in the relugolix combination therapy group in both the adenomyosis and overall LIBERTY 1/2 populations (Al-Hendy *et al.* 2021, Stewart *et al.* 2023, Catherino *et al.* 2025), as assessed by changes in the UFS-QOL score.

### Conclusion

In summing up, Catherino *et al.* (2025) stated that the findings of this *post hoc* analysis show no loss of efficacy in the treatment of uterine fibroids in women with concomitant adenomyosis.

### Limitations

Diagnosis of disease in the LIBERTY population may be subject to bias, given that adenomyosis was determined on the basis of retrospective evaluation of TVUS images, rather than MRI. Furthermore, baseline ultrasounds were not specifically done to identify adenomyosis (Catherino *et al.* 2025). In fact, adenomyosis may be overlooked in case of large fibroids, as shadowing may partially or completely obscure the disease (Van den Bosch *et al.* 2015, Cunningham *et al.* 2018, Harmsen *et al.* 2022, 2023).

It is also unfortunate that the results of relugolix without ABT (particularly its impact on volume) at week 12 were not communicated. Indeed, patients under relugolix alone were switched to relugolix with ABT at week 12, possibly explaining why the decrease was only 24.4% at week 24, similar to that observed in the combined therapy group.

In a retrospective study, Yamanaka *et al.* reported a reduction of 43% in uterine volume and 61% in adenomyotic lesions in 20 women with adenomyosis and concomitant fibroids after therapy of 16 weeks with 40 mg relugolix QD. This demonstrates the efficacy of high doses of relugolix without ABT (Yamanaka *et al.* 2023). It should nevertheless be noted that, in some countries, relugolix is only available in combination with ABT, so its administration should be evaluated in case of high body mass index or thromboembolic risks.

#### Linzagolix

*Post hoc* analysis of PRIMROSE 1/2 trials was not scientifically appropriate for evaluation of efficacy in women with uterine fibroids and concomitant adenomyosis. In the protocol, baseline ultrasounds were not systematically performed to detect adenomyosis at the start (Donnez *et al.* 2022*b*, Donnez *et al.* 2025*a*,*b*). To avoid any possible bias and limitations caused by *post hoc* testing and insufficiently defined criteria, it was decided to initiate a prospective, exploratory, open-label, single-arm pilot study in eight women with diffuse adenomyosis (Donnez *et al*. 2022*a*). This trial would determine the efficacy of a once-daily regimen of 200 mg linzagolix for 12 weeks, followed by a further 12 weeks on 100 mg and then 12 more weeks without therapy to investigate recurrence of symptoms. Diagnosis of diffuse adenomyosis was confirmed by MRI according to the classification of Bazot & Daraï (2018).

##### HMB

All patients reported HMB at baseline. Amenorrhea, defined as no uterine bleeding for at least 35 days, was recorded in all women by 12 weeks, and the mean time from treatment initiation to the start of amenorrhea was 21.3 ± 13.4 days (median: 22 days; range: 4–43 days) (Donnez *et al.* 2022*a*). At week 12, the patients were switched to the lower dose (100 mg linzagolix) and the majority were still amenorrheic at week 24. By week 36, however, after 12 weeks without therapy, all the women were experiencing HMB recurrence (Donnez *et al.* 2023).

##### Uterine volume

**Figure 1:**
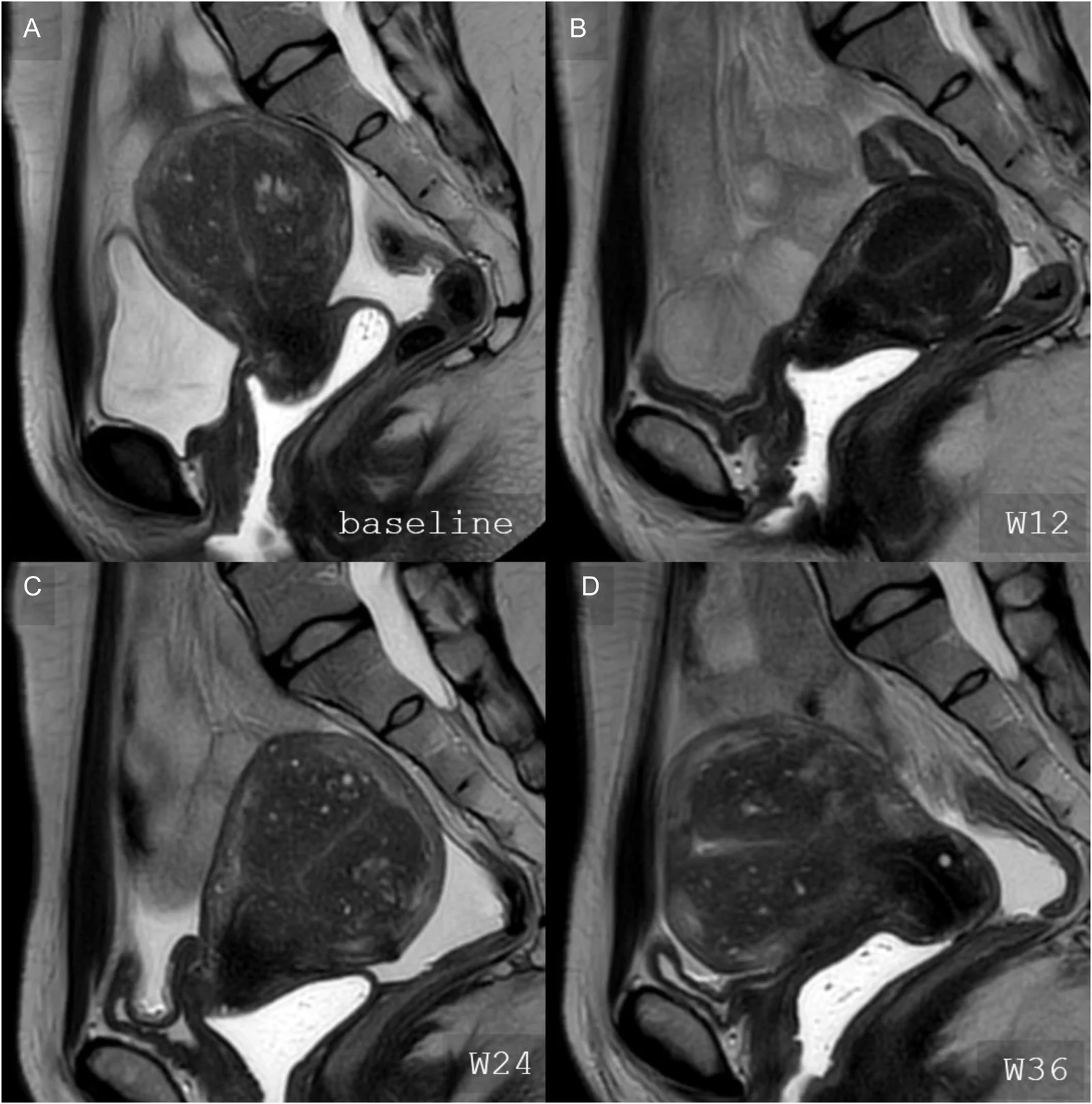
Patient 1. (A) MRI showing an enlarged uterus with diffuse and disseminated adenomyosis at baseline. (B) After 12 weeks of GnRH antagonist therapy (200 mg linzagolix, QD), a significant reduction was observed in uterine size and adenomyotic lesions. (C) The patient was then switched to 100 mg/day linzagolix, causing some regrowth, but the volume remained smaller than at baseline. (D) By week 36 (12 weeks after the end of therapy), uterine volume values returned to baseline values.

**Figure 2:**
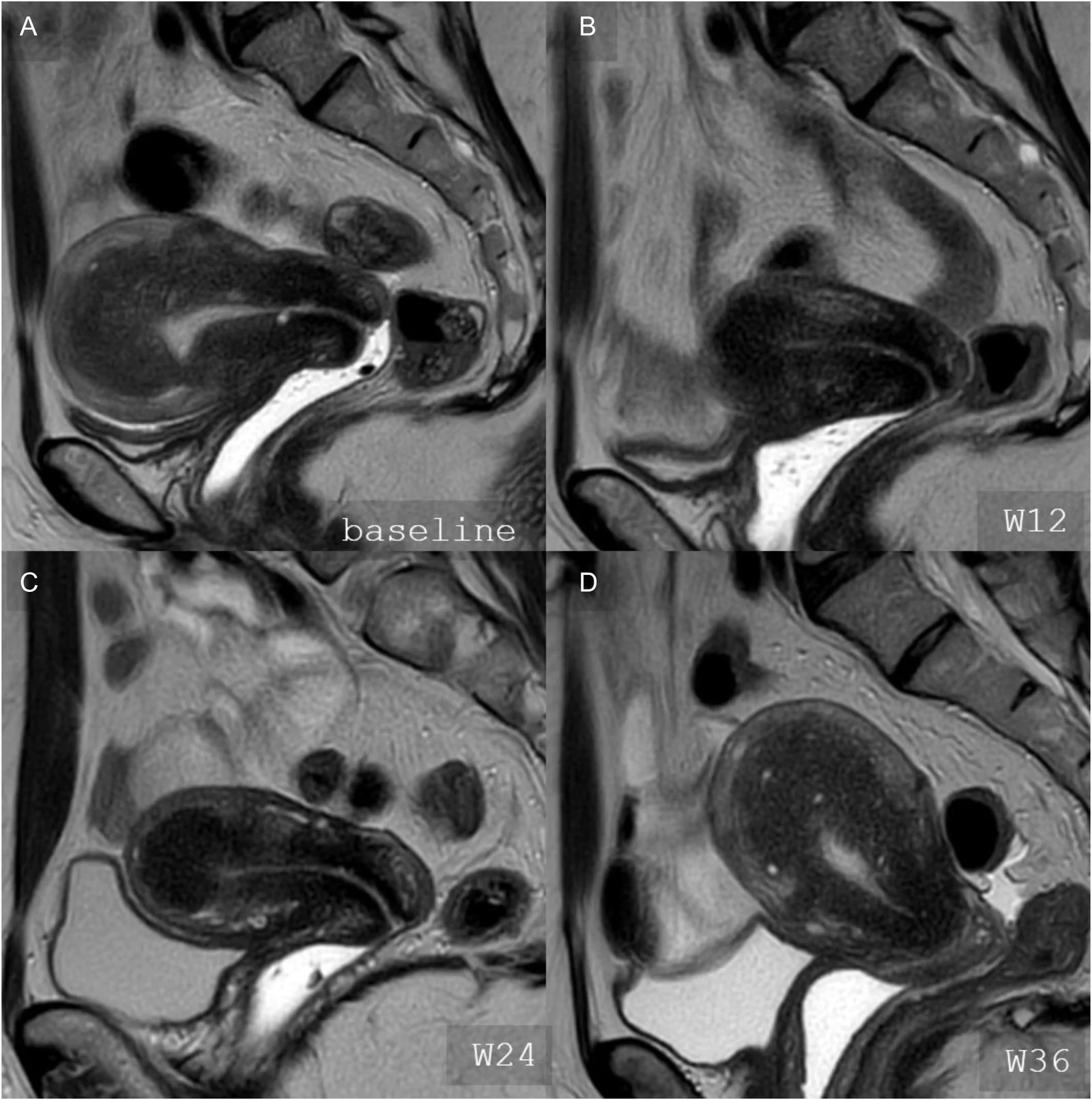
Patient 2. (A) MRI showing an enlarged uterus with diffuse and disseminated adenomyosis at baseline. (B) After 12 weeks of GnRH antagonist therapy (200 mg linzagolix, QD), a significant reduction was observed in uterine size and adenomyotic lesions. (C) The patient was then switched to 100 mg/day linzagolix, causing some regrowth, but the volume remained significantly smaller than at baseline. (D) By week 36 (12 weeks after the end of therapy), uterine volume values returned to baseline values.

The mean ± standard deviation of uterine volume was 333 ± 250 cm^3^ at baseline. By 12 weeks, MRI showed that it had dropped to 159 ± 95 cm^3^, yielding a mean reduction of 55% (*P* < 0.001). By week 24, the volume was 204 ± 126 cm^3^ (*P* = 0.0057), corresponding to a mean 32% decrease. In 5 out of 8 women, uterine volume remained similar between 12 and 24 weeks, while in the other 3, there was some increase between 12 and 24 weeks, but it was still below baseline at 24 weeks (Donnez *et al.* 2021*b*, 2022*b*). By week 36, the uterine volume was 377 ± 298 cm^3^, similar to baseline values (Figs 1 and 2) (Donnez *et al*. 2023). Serum E2 was fully suppressed during the first 12 weeks, with median serum E2 concentrations at 12 pg/mL by week 4 and maintained up to week 12, explaining the significant decrease in uterine volume. After patients were switched to 100 mg/QD linzagolix, the median serum E2 levels rose to around 35 pg/mL in the so-called optimal therapeutic zone according to Barbieri (1998). By week 36 (12 weeks after the end of therapy), E2 levels had returned to values observed in normal ovulatory cycles (Donnez *et al.* 2023).

Junctional zone thickness was 29 ± 12 mm at baseline, 19 ± 12 mm at 12 weeks and 23 ± 11 mm at 24 weeks, corresponding to mean 38 and 20% reductions from baseline. By week 36, it was back at 27 ± 18 mm (Donnez *et al.* 2021*b*, 2022*a*).

##### Pain and quality of life

Women with uterine fibroids and coexisting adenomyosis are significantly more likely to experience worse symptom severity and health-related quality of life than those without concomitant adenomyosis at baseline, as assessed by the UFS-QOL questionnaire. Mean global pelvic pain, dysmenorrhea, non-menstrual pelvic pain, dyspareunia and dyschezia scores all decreased over time. In terms of pelvic pain, mean ± SD scores were 8.4 ± 1.1 at baseline, 2.4 ± 3.4 at 12 weeks (*P* = 0.0035) and 0.6 ± 0.7 at 24 weeks (<0.0001), establishing that efficacy was maintained when patients were switched to 100 mg linzagolix QD (Donnez *et al.* 2021*b*). In the majority of cases, however, symptoms returned to baseline by week 36. Improvement in quality of life was evidenced by marked decreases in all EHP-30 domain scores (pain, control, powerlessness, emotional well-being, social support and self-image) at 12 and 24 weeks (Donnez *et al.* 2021*b*).

### Conclusion

The results of this study confirm that linzagolix administered to women with severe symptomatic adenomyosis at a high dose for 12 weeks, followed by a lower maintenance dose for a further 12 weeks, is effective. This protocol was found to substantially reduce uterine volume, lessen uterine bleeding, mitigate pain symptoms and enhance quality of life at 12 and 24 weeks. Nevertheless, as discussed in a recent paper, the rapid return to baseline values for uterine volume and bleeding unfortunately indicates that modified regimens are needed for long-term therapy (Donnez *et al.* 2023, Donnez & Dolmans 2025*c*). An initial course of 200 mg/QD linzagolix for 12 weeks and further treatment with either 100 mg/QD linzagolix or 200 mg/QD linzagolix with ABT should be investigated for long-term management.

**Figure 3:**
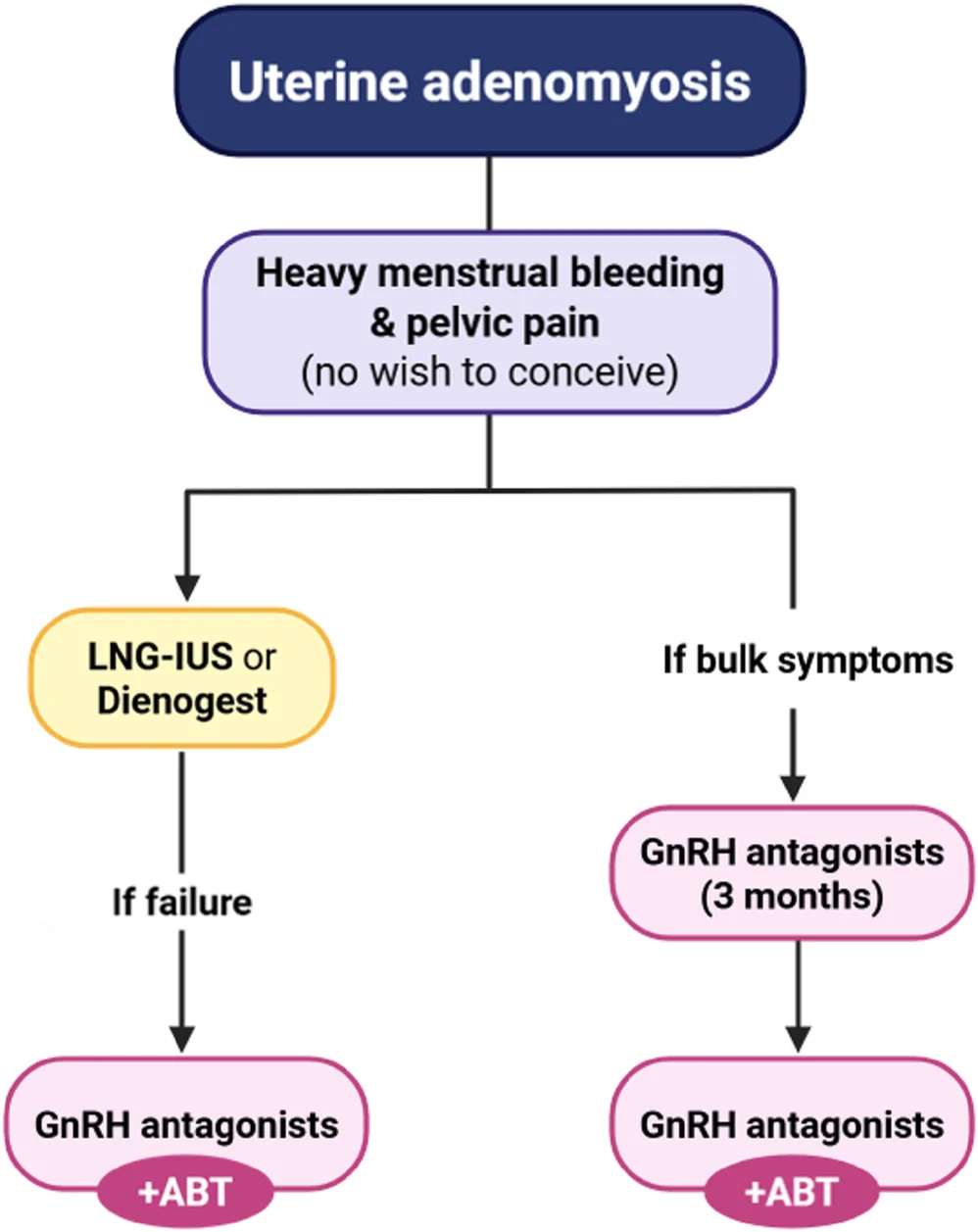
Proposed algorithm for uterine adenomyosis-related HMB/pain. As a first-line therapy, dienogest or the LNG-IUS should be considered. However, in case of failure or progesterone resistance, oral GnRH antagonists with ABT could be proposed as a long-term therapy. In case of an enlarged uterus with associated bulk symptoms, oral GnRH antagonists without ABT could be prescribed for 12 weeks to significantly decrease uterine volume, followed by long-term oral GnRH antagonists with ABT.

For uterine adenomyosis-related infertility, oocytes can be collected and vitrified before medical therapy with the 200 mg dose, as recently described (Donnez *et al.* 2023) (Fig. 3). After 12 weeks, when volume reduction is maximal, therapy can be stopped and embryos can be transferred 2–3 weeks later. Indeed, restoration of ovarian steroid secretion is very fast, as borne out by the onset of the first menstrual bleed after the end of therapy (mean: 31 days) (Donnez *et al.* 2025*a*,*b*).

One advantage of using different doses of linzagolix is that E2 suppression can be modulated by changing doses (such as switching from 200 to 100 mg in the reported study) (Donnez *et al.* 2022*a*) to reduce hypoestrogenic side effects, while maintaining efficacy in terms of control of bleeding, pain and quality of life.

### Limitations

Under linzagolix therapy (200 mg QD), BMD loss was observed from baseline to 24 weeks (Donnez *et al.* 2021*b*, 2022*a*, 2025*a*,*b*). This is the price to pay if a significant reduction in uterine volume is the goal. It should nevertheless be emphasized that very recent publications reported some degree of recovery of BMD when treatment was stopped or when ABT was added at the end of therapy with 200 mg linzagolix alone (Donnez *et al.* 2025*a*,*d*).

Although this study was prospective and analyzed data from women with proven adenomyosis documented by MRI, it should be acknowledged that the number of cases was limited and randomized controlled trials are needed to corroborate and validate this concept**.** Moreover, the evaluation was restricted solely to cases of diffuse adenomyosis.

## Discussion and conclusion

With improvements in TVUS and MRI, it is now possible to more reliably identify adenomyosis through imaging and hence diagnose affected women sooner. It has become apparent that visible features of adenomyosis can be spotted in as many as 35% of reproductive-age girls and women up to 30 years of age with no history of pregnancy or uterine surgery (Pinzauti *et al*. 2015, Vannuccini *et al.* 2018, 2025, Exacoustos *et al.* 2022, Mishra *et al.* 2023). In the trials described here, the prevalence of concurrent adenomyosis in women with uterine fibroids was about 16%.

Symptoms of adenomyosis include abnormal uterine bleeding, particularly HMB, dysmenorrhea, dyspareunia, infertility and a variety of disorders associated with pregnancy. According to Struble *et al.* (2016), at least 30% of women with adenomyosis are asymptomatic. However, this rate could well be an underestimation, as dysmenorrhea, dyspareunia and pelvic pain are not always recorded by clinicians (Catherino *et al.* 2025).

The principal objective of treating adenomyosis is symptom management. Treatment requires a long-term management plan, as the disease often has a chronic negative impact on quality of life until menopause is reached (Vannuccini *et al.* 2018), but data on medical therapy for adenomyosis are limited. Progestogens and COCs are not very effective (Donnez *et al.* 2021*a*, Vannuccini *et al.* 2025). Indeed, progesterone resistance in adenomyotic endometrium and stroma is actually considered another characteristic of uterine adenomyosis (Mehasseb *et al.* 2011, Liu *et al.* 2016, Donnez *et al.* 2020*b*, Guo 2020, Habiba *et al.* 2020). Decreased expression of progesterone receptor B in adenomyotic lesions has been reported to be the underlying cause of progesterone resistance and is thought to be epigenetically regulated (Nie *et al.* 2021, Bulun *et al.* 2023). No specific drug is currently approved for the treatment of adenomyosis, but a number of hormones are often used off-label to alleviate symptoms and have demonstrated some degree of efficacy, as reported in Table 1. These drugs should be regarded as a first-line therapy in the management of uterine adenomyosis-related HMB and pain. However, there is clearly a widespread demand for improved long-term medical therapies for adenomyosis and oral GnRH antagonists have emerged as a potential alternative to GnRH agonists by allowing dose-dependent control of E2 levels (Donnez *et al.* 2017, Donnez & Donnez 2020*a*, Donnez *et al*. 2020*b*). Apart from their unique capacity to modulate E2 suppression, another advantage of orally active GnRH antagonist over GnRH agonist depot formulations is the absence of the flare-up effect, hence avoiding initially worsening symptoms and rapid reversibility. Several studies have recently demonstrated that oral, nonpeptide GnRH antagonists are effective at treating endometriosis-associated pain (Taylor *et al.* 2017, Osuga *et al.* 2021, Giudice *et al.* 2022, Donnez *et al.* 2024*b*). Since all pathways involved in the pathogenesis of adenomyosis also feature estrogens in a major role, the same therapy could be applied to symptomatic adenomyosis (Donnez *et al.* 2022*a*,*b*).

## Future perspectives

The goal of this narrative review was to evaluate the place of this new class of oral medication. In addition to HMB, women with associated adenomyosis may experience pelvic pain because of swelling of endometrial islands confined within the myometrium (Donnez *et al.* 2019, Yamanaka *et al.* 2023). These related symptoms of bleeding and pain can have a major impact on women’s quality of life, psychological and social well-being and overall health. All three oral antagonists (elagolix, relugolix and linzagolix) were able to control the disease and uterine adenomyosis-related HMB and pain and enhance quality of life, allowing many patients to avoid hysterectomy and keep their uterus. Indeed, preserving the uterus in case of adenomyosis remains a key challenge (Di Spiezio Sardo *et al.* 2025) in the knowledge that 80% of women with symptomatic adenomyosis in the USA actually undergo hysterectomy (Catherino *et al.* 2025). The available evidence is promising, but should clearly be confirmed by further clinical trials. It should also be stressed that these antagonists are still used off-label for adenomyosis.

Interestingly, the significant reduction in uterine volume achieved in women with adenomyosis through treatment with oral GnRH antagonists without ABT (elagolix (300 mg BID)), relugolix (40 mg QD) or linzagolix (200 mg QD)) may also yield enhanced fertility outcomes by decreasing uterine volume and lesion severity prior to IVF (Borini & Coticchio 2020, Catherino *et al.* 2025, Donnez & Dolmans 2025*c*). The studies discussed here demonstrate that high doses of oral GnRH antagonist should be given without ABT if a significant reduction in volume is sought. However, larger corroborative studies are required to establish definitive conclusions on fertility outcomes. Data reported in the studies of Catherino *et al.* (2025), Donnez *et al.* (2023) and Donnez & Dolmans (2025*c*) cannot simply be extrapolated to women suffering from uterine adenomyosis-related infertility.

Two algorithms can be proposed for further evaluation, taking into account the first-line medical therapies. In case of uterine adenomyosis-related HMB/pain (Fig. 3), dienogest or the LNG-IUS containing 52 mg of levonorgestrel should be considered as a first-line therapy, but in the event of failure or progesterone resistance, oral GnRH antagonists with ABT could be proposed as a long-term therapy. In case of an enlarged uterus with associated bulk symptoms, oral GnRH antagonists without ABT could be prescribed for 12 weeks to significantly decrease uterine volume.

**Figure 4:**
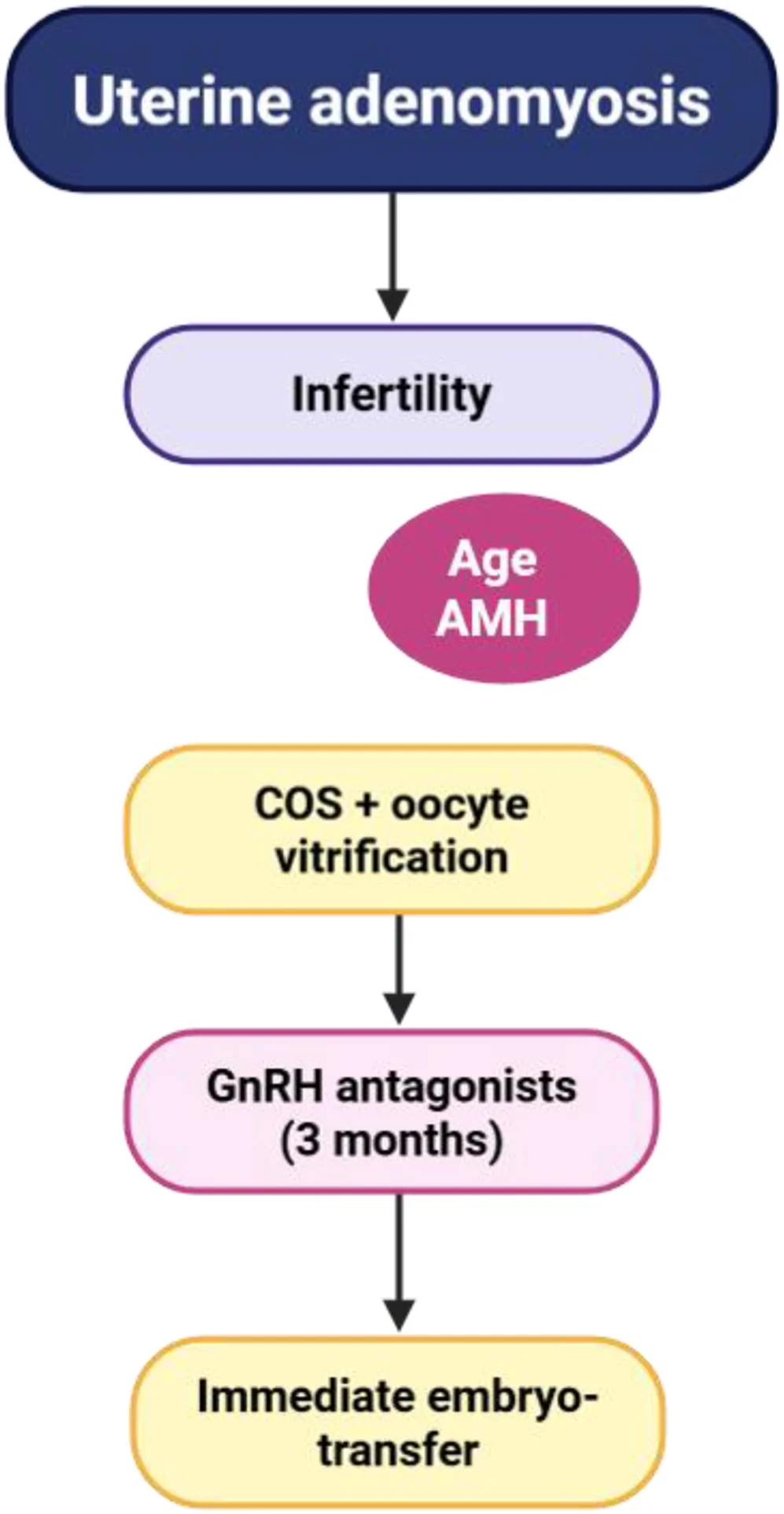
Proposed algorithm for uterine adenomyosis-related infertility. In case of infertility, and depending on age and anti-Müllerian hormone levels, controlled ovarian stimulation and oocyte pick-up followed by oocyte vitrification should be performed before any treatment with GnRH antagonists. GnRH antagonists without ABT should ideally then be given for 12 weeks, looking to reduce uterine volume, junctional zone thickness and the severity of lesions. Two weeks after the end of therapy, embryo transfer should be performed, as recovery of ovarian function is very fast.

In case of infertility (Fig. 4), controlled ovarian stimulation and oocyte pick-up followed by oocyte vitrification should ideally be performed before treatment with GnRH antagonists without ABT, looking to decrease uterine volume, junctional zone thickness and the severity of lesions. Two weeks after the end of therapy, embryo transfer can be performed, as recovery of ovarian function is very fast. Depending on symptoms (bleeding, infertility or both), age and reproductive status, randomized controlled trials need to be conducted to validate the therapeutic impact of oral GnRH antagonists in adenomyosis patients. The two algorithms provide the basis upon which protocols for clinical trials can be determined. In cases of adenomyosis-related uterine bleeding, comparisons should be made between placebo, dienogest, the LNG-IUS and GnRH antagonists. For uterine adenomyosis-related infertility, the efficacy of pre-IVF therapy (as shown in Fig. 4) with GnRH antagonists vs a placebo should be appraised.

Moreover, secondary prevention should not be overlooked (Vercellini *et al.* 2025). The riskiest period for lesion formation is from early adolescence to approximately 25 years of age, after which most lesions stabilize (Vercellini *et al.* 2025). Secondary prevention aims to curb the impact of disease by detecting and treating the condition or injury as soon as possible to halt or slow its progression. In their recent review, Vercellini *et al.* (2025) stressed that this ambitious goal focuses on intercepting adenomyosis and endometriosis at their earliest stage of development, preferably upon disease onset, and promptly suppressing regular ovulatory cycles by establishing a mildly estrogenic but stable hormonal milieu until patients wish to conceive. The place of a low-dose oral antagonist such as linzagolix (75 or 100 mg), which induces a dose-dependent reduction in estradiol levels in the therapeutic range without significant side effects, should at least be explored. It must nevertheless be pointed out that ovulation may still occur during low-dose therapy and that this treatment should be reserved for women with no sexual activity or those committed to using double-barrier contraception correctly and systematically.

In conclusion, GnRH antagonists may just offer that glimmer of hope that so many women are seeking (Donnez & Dolmans 2025*c*).

## Declaration of interest

JD was a member of the scientific advisory board of ObsEva and Preglem until 2023 and reports consulting fees from ObsEva, Gedeon Richter and Theramex. CMB was a member of the independent data monitoring board for the PRIMROSE trials and the advisory board for SPIRIT 1 and 2 trials. He was also the chair of the ESHRE endometriosis guideline committee. Consulting fees from Myovant and Theramex went to the University of Oxford. FP received consulting fees and honoraria for lectures from Theramex. AWH received consulting and lecture fees from Roche Diagnostics, Gesynta and Theramex, received grant funding from the EU, UKRI, NIHR, CSO, Wellbeing of Women and Roche Diagnostics and has a patent on chronic pelvic pain treatment owned by the University of Edinburgh. He was also on the data safety monitoring board for PANDA, as well as a trustee and medical advisor to Endometriosis UK and a specialty advisor to the Scottish government’s Chief Medical Officer for Obstetrics and Gynaecology. He is also a co-Editor-in-Chief of *Reproduction & Fertility* and was not involved in the review or editorial process for this paper, on which he is listed as an author. SB received consulting fees and honoraria for lectures from Theramex. SPR reports consulting fees and honoraria for lectures from Gedeon Richter and Theramex. FCH reports consulting fees and honoraria for lectures, presentations or educational events from Theramex and Gedeon Richter and was a member of the data safety monitoring board for Organon. HT received grants from Abbvie and consulting fees from ObsEva and Gedeon Richter, has a patent on endometriosis biomarkers owned by Yale University and was a past president of the American Society for Reproductive Medicine (ASRM). MP was a principal investigator in the ObsEva-sponsored EDELWEISS trials. EB is an employee at Theramex, London. CAS has no conflict of interest. MMD received fees for lectures from Gedeon Richter and Theramex.

## Funding

Grant 5/4/150/5 was awarded to MMD by the FNRS.

## Author contribution statement

JD, MMD, EB and HST were involved in conceptualization and methodology. JD and MMD wrote/prepared the original draft. JD, OD, HST, FP, AWH, CMB, SPR, MR, EB, CAS and MMD performed data analysis and critically reviewed the manuscript. All authors have read and agreed to the final version of the manuscript.
